# Nutrient timing with low carbohydrate diets

**DOI:** 10.1186/1550-2783-8-S1-P7

**Published:** 2011-11-07

**Authors:** Pat Jacobs

**Affiliations:** 1 Superior Performance, Miami FL, USA

## Background

The health and weight control benefits of low carbohydrate diets are well established. Likewise, nutrient timing has been shown to effectively enhance exercise performance. However, there exists an apparent conflict between these two dietary strategies. In fact, many authorities consider high glycemic carbohydrates to be a necessary component of nutrient timing and there is no place in athletic training or competition for low carbohydrate diets.

## Low carbohydrate diets

Various low carbohydrate diets have been shown to provide beneficial changes in body mass, lipid profiles and other health risk factors. Recent evidence indicates that diets with lower glycemic index carbohydrates and increased protein provide greater weight loss and maintenance of the reduced weight as compared with high glycemic and low protein diets. Insulin release is lower with lower blood glucose levels, thereby reducing fatty acid metabolism and storage. High intake of high glycemic response carbohydrates is associated with increased levels of insulin resistance which appears to be the common link between obesity and metabolic syndrome.

## Nutrient timing

Nutrient timing is generally regarded as a nutritional strategy in which precise amounts of particular nutrients are delivered at precise time points, relative to exercise, in order to enhance performance or training effects. This somewhat general definition has been operationally limited by many to diets that utilize high glycemic carbohydrates prior to, during, and/or following exercise. These carbohydrates are considered vital as they provide an energy source as well as inducing increased insulin levels. As insulin directly influences the production of nitric oxide, vascular musculature is relaxed and circulation into the capillary beds of exercising muscles is increased. Carbohydrates, in particular higher glycemic carbohydrates, supply these critical benefits.

## Low carbohydrate nutrient timing

The basic model of low carbohydrate nutrient timing applies specific proven micronutrients for enhanced exercise performance rather than relying on the ingestion of sugar and the subsequent insulin responses. First, reduced carbohydrate intake produces reduced insulin responses which shifts the metabolism to fatty acid utilization. Secondly, various nutritional components can provide additional energy sources and/or produce increased nitric oxide production with subsequent vasodilation. Items such as creatine and beta alanine can influence energy levels by affecting energy replenishment and acting as an anaerobic buffer. Branched chain amino acids provide a third energy source without which muscle tissue may be consumed with intense exercise. Various micronutrients can increase muscle blood flow to some degree. In particular, glycine proprionyl l-carnitine (GPLC) has been shown to dramatically increase nitric oxide synthesis in response to exercise stresses and to significantly increase exercise performance with reduced production of lactate.

## Conclusions

The limited research in the area suggests that some athletes can train and compete in certain settings successfully with relatively low intake of dietary carbohydrates. It has been shown that pre-workout supplements containing common ingredients such as creatine, beta alanine, branched chain amino acids can substantially enhance exercise performance without ingestion of additional carbohydrates. Controlled clinical trials are needed to examine the effectiveness of nutrient timing with a low carbohydrate diet in various sports settings.

